# Acceptability of Suubi+Adherence intervention to improve ART adherence in Southern Uganda: A qualitative analysis

**DOI:** 10.1371/journal.pone.0347112

**Published:** 2026-04-24

**Authors:** Josephine Nabayinda, Ozge Sensoy Bahar, Phionah Namatovu, Proscovia Nabunya, Samuel Kizito, Anita Kabarambi, April Thames, Fred M. Ssewamala

**Affiliations:** 1 Washington University in St. Louis Brown School, St. Louis, Missouri, United States of America; 2 New York University Silver School of Social Work, New York, New York, United States of America; 3 International Center for Child Health and Development (ICHAD), Masaka Office, Masaka, Uganda; 4 University of California, Los Angeles, Department of Psychiatry and Biobehavioral Sciences, Los Angeles, California, United States of America; Institute of Tropical Medicine / University of Antwerp, BELGIUM

## Abstract

In Uganda, 150,000 young people were living with HIV by the end of 2023. While Antiretroviral therapy (ART) is effective in reducing HIV transmission, youth in SSA, including in Uganda, face greater challenges with ART adherence compared to adults. Poverty-focused interventions have been recommended to improve adherence and viral load suppression. In this manuscript from Suubi+Adherence-Round 2 study, we explored the acceptability of Suubi+Adherence, a combination intervention aimed at improving ART adherence among adolescents living with HIV in Uganda that was tested in a six-year longitudinal study called Suubi+Adherence (2012–2018). We conducted semi-structured in-depth interviews with 36 youths who participated in the Suubi+Adherence intervention, which comprised matched savings accounts, mentorship, financial management, and business development training. Interviews explored participants’ motivations for joining, their experiences with the intervention, and the facilitators and barriers to intervention attendance. Informed by the Theoretical Framework of Acceptability, data were analyzed using thematic analysis and Dedoose software. Our results showed that the primary motivation for participation was the opportunity to learn about savings and income-generating activities. Participants also appreciated the Wisepill -an Electronic Monitoring Device, which reminded them to take their medication. Key facilitators for intervention attendance included transport reimbursements, family support, and content relevance. Challenges included long travel distances and associated costs, session timing conflicts with household responsibilities, and other personal obligations. Despite these challenges, participants highlighted the practical relevance of the intervention content, which aligned with their real-life experiences and needs. Our findings offer valuable insights into the acceptability of a combination intervention aimed at improving ART adherence among youth. These results have important implications for HIV treatment programs and policy in Uganda, particularly given the country’s high HIV prevalence. The parent randomized clinical trial is registered in the clinical trials database (NCT03307226).

## Introduction

Sub-Saharan Africa (SSA) bears the highest HIV prevalence globally, with two-thirds of the 3.4 million youths aged 15–24 living with HIV worldwide residing in this region. [1]. Uganda, one of the hardest-hit countries in SSA, reports unprecedented numbers of young people living with HIV (YLHIV). As of 2023, 150,000 young people aged 15–24 were living with HIV in the country, with this age group accounting for 70% of all new HIV infections [2]. Effective Antiretroviral therapy (ART) has proven to be highly effective in reducing the risk of HIV transmission, as well as promoting virologic suppression, immune reconstitution, and decreased morbidity [3,4]. In addition to its clinical benefits, ART offers important social and economic advantages, such as improved quality of life [5,6], decreased stigmatization and discrimination associated with HIV [7], and increased productivity and workforce participation [8,9]. Conversely, poor adherence can lead to viral rebound, disease progression, and drug resistance, compromising clinical outcomes [10].

The effectiveness of ART in achieving virologic suppression is heavily dependent on adherence [10,11]. When compared to adults, YLHIV in SSA are at a higher risk of poor HIV treatment adherence [12], which has been strongly associated with adverse treatment outcomes, including higher rates of virologic failure, drug resistance, and suboptimal CD4 recovery [13,14]. For instance, only 20% of YLHIV achieve 100% adherence to ART over 6 months, compared to over 40% of adults [15]. Lower levels of adherence to HIV medication among YLHIV are influenced by a range of individual, psychosocial, treatment-related, structural, and economic factors. Individual and psychosocial barriers include limited caregiver supervision, lack of HIV status disclosure within the family, academic pressure, small support networks, negative attitudes toward HIV, and misperceptions or a lack of awareness about the disease and its treatment [16,17]. Treatment-related factors, such as high pill burdens, adverse side effects, fear of HIV status disclosure, and difficulties during the transition from pediatric to adult HIV care, also hinder consistent treatment adherence [18,19]. Additionally, economic challenges, including limited access to food and high transportation costs, further restrict access to HIV care among YLHIV, often leading to inconsistent medication adherence and poor treatment outcomes [17,20].

Poverty-focused interventions have been advocated to improve adherence and enhance viral load suppression among people living with HIV, including YLHIV [21]. However, previous studies integrating such interventions have used conditioned cash incentives for adherence, often resulting in low success rates in improving adherence or no difference in viral suppression [22,23]. Furthermore, there remains a dearth of literature on the acceptability and effectiveness of these interventions in HIV prevention and treatment, particularly in low- and middle-income countries (LMICs) [24]. A recent systematic review of peer-reviewed studies assessing intervention acceptability among African youth (aged 10–24) focused primarily on HIV-related or sexual and reproductive health outcomes [24]. While informative, these interventions were either standalone or explored only a single component of combination interventions, failing to assess acceptability holistically. Other studies in similar settings have been limited by either gender-specific sampling or non-HIV populations [25,26]. In the context of HIV prevention, existing acceptability studies predominantly examine pre-exposure prophylaxis (PrEP) uptake, emphasizing facilitators and barriers [27,28].

Given these gaps, more studies are needed to assess the acceptability of combination interventions -particularly those integrating economic empowerment and addressing multiple HIV risk factors. In this study, we explore the acceptability of a combination intervention named Suubi+Adherence (Suubi, meaning “hope” in Luganda, the local language of the study area), which tested the impact of the Suubi+Adherence intervention on improving adherence among adolescent boys and girls in Uganda.

## Methods

### Overview of the Suubi+Adherence study

The original *Suubi+Adherence* study (2012–2018), a cluster-randomized clinical trial (NCT01790373), funded by the Eunice Kennedy Shriver National Institutes of Child Health and Human Development (NICHD, R01HD074949; PI Fred Ssewamala), was designed to evaluate the impact and cost-effectiveness of a family economic empowerment (FEE) intervention in improving HIV treatment adherence among adolescents living with HIV (ALHIV) in Uganda. The study began with a total of 728 ALHIV from 40 clinics, however, after disqualifying one clinic—following its closure by the district health officials for lack of an operational license—the study remained with a total of 702 ALHIV (ages 10–16 at enrollment) from 39 health clinics (344 participants across 19 clinics in the bolstered standard care condition and 358 participants across 20 clinics in the intervention arm). The study was conducted in Southern Uganda, a region with an HIV prevalence of 11.7%, more than double the national average of 5.4% [2]. Eligible participants were adolescents who were 10–16 years old, living with HIV, aware of their HIV status, living in a family setting, and receiving ART from one of the collaborating clinics. Participants were randomized at the clinic level into two study conditions: 1) **Bolstered standard of care (BSOC)**: As part of routine care across all public HIV care clinics, all ALHIV receive clinical and psychosocial support according to Uganda Ministry of Health’s guidelines [29]. However, the psychosocial support is highly variable in frequency and quality across clinics. Therefore, irrespective of the study group, all participants in the study received BSOC, which consisted of eight adherence-support information sessions using print cartoons to portray adherence topics in a relatable manner adapted from the VUKA intervention for ALHIV in South Africa [30]; and 2) **FEE Intervention + BSOC group**, a two-year intervention which included three components: a) **Child Development Account (CDA)**, matched at a rate of 1:1 up to USD 10/month) and jointly held by adolescents and caregivers; b) **Twelve mentorship sessions** led by peer mentors focused on goal-setting, financial literacy, and healthy behavioral decision making; and c) **Four one-hour microenterprise workshop group sessions** intended on providing financial management and income-generating activity training, including crop and animal husbandry, tailoring, basket weaving, and knitting (see Ssewamala et al [31] for more details on the study design). The intervention has been found effective in improving ART adherence, enhancing academic achievement, and addressing mental health challenges among adolescents [32–35]

The original Suubi+Adherence study was followed by **Suubi+Adherence-Round 2** (2020–2025), also funded by NICHD (R01HD074949; MPIs Ssewamala, Sensoy Bahar, Nabunya) that aimed at examining longitudinal patterns of HIV treatment adherence among participants from the original cohort as they transitioned into young adulthood (see Ssewamala et al., 2021 [31] for more details). Of the 702 participants in the original study, 572 participants (n = 293 in the intervention arm across 20 clinics) were re-consented to participate in Suubi+Adherence Round 2. As part of Round 2, a qualitative component was included to explore: a) multi-level factors affecting participants’ maintenance of intervention benefits since Suubi+Adherence intervention initiation (prospectively); and b) participants’ experiences with the intervention (retrospectively), including multi-level factors that may have influenced their engagement with the program, as well as their decision-making regarding ART adherence. In this study, we focus on participants’ experiences with the intervention.

### Qualitative sampling for the Suubi+Adherence Round 2 study

A stratified purposeful sampling approach was used to select a qualitative subsample of 40 participants from the intervention arm (N = 293). Stratification was conducted across outcomes—including sexual risk-taking, mental health, and matched savings—to ensure variation in the qualitative sample. The first stratification was based on the participants’ sexual risk-taking behaviors. Specifically, we classified the participants into those with or without a sexually transmitted infection (STI), following laboratory confirmation. Five participants were randomly selected from each stratum.

Similarly, we stratified the participants based on the participants’ mental health functioning (into the lowest and highest quartiles) at the 72-month follow-up. Mental health was assessed using three measures: the Tennessee Self-Concept Scale [36], Beck’s Hopelessness Scale [37], and the Center for Epidemiological Studies–Depression Scale (CES-D) [38]. A composite score was generated from all three scales, and participants were randomly selected from the lowest (n = 5) and highest (n = 5) quartiles.

Finally, monthly bank statements from the original Suubi+Adherence study were used to stratify participants into the lowest and highest quartiles of savings, from which 10 participants were randomly selected (five participants from each stratum). We also stratified participants who did not have savings into those who did not open an account and those who opened an account but did not save. We then randomly selected five participants from each group. Across all three outcomes, 40 participants were selected from the intervention arm. A total of 36 participants completed the interviews.

This sampling strategy, informed by key outcomes, was utilized to examine differences and commonalities in intervention experiences among participants who benefited to varying degrees from the intervention.

### Qualitative data collection

The semi-structured interview guide development was led by co-authors with formal training and expertise in qualitative research, in close collaboration with the research team. The guide was informed by the aims of the Suubi+Adherence Round 2 study, prior qualitative work conducted within the Suubi program, and questions used in earlier studies assessing intervention acceptability [25,26,39]. The guide was translated into Luganda and reviewed by bilingual team members for accuracy and contextual appropriateness. To ensure cultural and age appropriateness, the semi-structured interview guide was pretested with a small group of youth of similar age to the study population. Pretesting assessed clarity, flow, comprehension, and relevance of the questions. Feedback from pretesting resulted in minor refinements to wording and sequencing to enhance comprehension and conversational naturalness.

Face-to-face semi-structured in-depth interviews were conducted after the completion of wave six quantitative data collection, between February 1st, 2022, and March 30th, 2022, approximately five years after intervention completion. The interviews focused on participants’ [1] experiences with the intervention, including each of its specific components; [2] key multi-level (individual, family, contextual, and program) influences on program participation; and [3] multi-level factors that may have influenced their decision-making and behaviors regarding ART adherence and overall mental health since the completion of the Suubi+Adherence study. The interview questions included: “Can you tell us a little bit about why you decided to participate in the Suubi+Adherence program?” “Can you share your experience with the program?” “Can you tell us about your experience attending the sessions?” “What information or skills covered in the sessions did you like, and what aspects you didn’t like?” “What factors made it easier for you to attend the sessions?” “What factors made it difficult for you to attend the sessions, and how did you overcome these barriers?” “How has participation in the program affected your adherence to medication?

The interviews were conducted in Luganda, the most widely spoken language in the study region. Research assistants fluent in both Luganda and English and trained by the Principal Investigators (PIs) with expertise in qualitative research conducted the interviews. Each interview, lasting between 30 and 97 minutes (mean = 57 minutes), was held in a private space with only the research assistant and participant present. All interviews were audio-recorded.

### Ethical considerations

Participation in the study was voluntary. The study was approved by Makerere University School of Public Health Research and Ethics Committee (Protocol # 210) in Uganda, Uganda National Council for Science and Technology (UNCST, SS, 2969), the Columbia University Review Board (AAAK3852; 2012–2017), and the Institutional Review Board at Washington University in St. Louis (IRB # 201704066; 2017-current) in the USA. For the original Suubi+Adherence study, all adolescent participants provided age-appropriate informed assent. In addition, written informed consent was obtained from the caregivers of the participants. The consenting and assent activities were conducted separately for the adolescents and caregivers to avoid potential coercion. Before enrollment in Suubi+Adherence-R2, participants from the original Suubi+Adherence study, all older than 18 years old at the time of Round 2, were re-consented. All the study staff received human subjects research training by completing the Good Clinical Practice (GCP) training and the Collaborative Institutional Training Initiative (CITI) ethics training.

### Qualitative data analysis

Interviews were first transcribed verbatim and then translated from Luganda to English by research assistants fluent in both languages. We employed inductive techniques for thematic analysis of the data [40,41]. Themes were initially identified and then broken down into smaller, more specific units until no further subcategories were necessary. The transcripts were independently coded by two research team members, and any disagreements were resolved through team discussions. The initial codes were reviewed and discussed with the first author during team meetings and reorganized when necessary, resulting in a final codebook used to code all transcripts. Once coding reliability was established, interviews were coded independently by two team members. The analysis, conducted by two authors, compared and contrasted themes and categories to identify similarities, differences, and relationships among the findings. To ensure rigor, we employed peer debriefing and maintained an audit trail [41,42]. The codes and findings were then presented to two members of the research team who were not involved in the data analysis, to evaluate the plausibility of the identified themes and related findings [43,44]. Pseudonyms are used throughout to protect participant confidentiality.

### Theory guiding the qualitative data analysis

We used Sekhon’s theoretical framework of acceptability (TFA) to guide our qualitative analysis [45]. According to TFA, acceptability is a multi-faceted construct that reflects the extent to which individuals delivering or receiving a healthcare intervention consider it appropriate, based on their anticipated or experienced cognitive and emotional responses. TFA consists of seven constructs: affective attitude, burden, perceived effectiveness, ethicality, intervention coherence, opportunity costs, and self-efficacy. Affective attitude refers to how an individual feels about the intervention, both before (anticipated) and after (experienced) participating. This construct captures the emotional response to the intervention, reflecting whether the individual finds it appealing or acceptable. Burden includes the perceived effort required to participate in the intervention (anticipated) and the actual effort expended during participation (experienced) in terms of time, effort, and inconvenience.

Perceived effectiveness refers to both how likely the intervention is expected to achieve its goals (anticipated) and how successful participants believe it has been in achieving those goals (experienced). Ethicality refers to how well the intervention aligns with the participant’s value system, i.e., whether the intervention feels morally acceptable and appropriate for the individual. Intervention coherence refers to how well participants understand the intervention. It captures whether the intervention makes sense to the participant and how well its purpose and process are communicated. Self-efficacy pertains to the participant’s self-confidence in their ability to perform the behaviors necessary for successful participation in the intervention. Finally, opportunity cost refers to the perceived value of what one must give up to engage in the intervention (anticipated) and the actual sacrifices made (experienced). This construct highlights the trade-offs participants consider, such as time, resources, or other activities, in deciding whether to participate in the intervention.

In this study, Sekhon’s TFA served as the conceptual framework guiding data analysis. Because acceptability is conceptualized in TFA as a composite of interrelated constructs, we examined these constructs to understand participants’ experiences with the Suubi+Adherence intervention, and relatedly acceptability of the intervention.

## Results

### Participant demographics

The mean age of participants (n = 36) was 20.19 years. Half of the participants (50%) were male. Fifty-eight percent were orphans (lost one or both parents), and 63% were not employed at the time of the interview. Additionally, 66% reported not being in a relationship. The number of people in each household varied from one to twelve, and 75% of participants were not attending school at the time of qualitative data collection. See Table 1 for details.

**Table 1:** Participant demographics in Suubi+Adherence-R2 qualitative sample.

Variable	Total sample (N = 36) % (n)
Age (Mean, SD)	20.19 (1.83)
Household size (Mean, SD)	4.28 (2.46)
*Gender*	
Male	50.0 (18)
Female	50.0 (18)
*Orphanhood status*	
Non orphan	41.7 (15)
Orphan	58.3 (21)
*Employment status*	
Currently employed	36.1 (13)
Not employed	63.9 (23)
*Marital Status*	
Single	66.7 (24)
Married	11.1 (4)
Cohabiting	22.2 (8)
*Education level*	
Not in school	75.0 (27)
Secondary	22.2 (8)
Vocational/Tertiary	2.8 (1)

### Overview of themes and subthemes in relation to the TFA

To fully assess the acceptability and appropriateness of the Suubi+Adherence intervention for the participants, we explored their decision-making processes, specifically their motivations about participating in the program before enrollment, as well as the barriers and facilitators to their attendance, and how they relate to the Theoretical Framework of Acceptability. Additionally, we sought their feedback on the relevance of the content and the aspects of the program they learned and had continued to use in their day-to-day life. Table 2 summarizes the themes in relation to the guiding theory.

**Table 2:** Overview of themes and subthemes in relation to the TFA.

Theme	Subtheme	Related TFA Construct
Motivation to participate	• Opportunity to learn • Opportunity to open bank accounts • Anticipation for support • Opportunity to start up income generating activities • Wise pill devices	Anticipated affective attitude Anticipated perceived effectiveness
Facilitators to program attendance	• Content learned • Support from family members • Transport refund • Sense of belonging • Snacks (cakes and sodas) • Staff conduct • Facilitators attitude • Proper/Good planning	Experienced opportunity cost Experienced burden
Barriers to program attendance	• Transport costs • Parental involvement • Timing of the sessions versus other commitments • Stigma and fear • Sickness	Experienced opportunity cost Experienced burden
Perceived impact	• Cartoon sessions • Adherence to medication • Self-protective behaviors • Wise pill devices • Improving relationships with people • Saving • Starting income generating activities	Experienced perceived effectiveness Intervention coherence Self-efficacy

### Motivation to participate in the program

The participants identified several reasons for their involvement, reflecting various aspects of the program. Some emphasized the opportunity to learn about different components, while others were particularly excited about the Wisepill devices, the chance to open bank accounts in their own names, and access to banking services as well as matched savings. For example, John was motivated by the program’s promise to open bank accounts for them, “*We were told that they were going to open up accounts for us, which would help me save some money that would help in the future. That motivated me to join the program.”*

Similar sentiments were echoed by Mary. Although she was not able to move forward with opening the account, she found it to be one of the components that excited her about joining the program, along with the matched savings and financial literacy sessions.

***Mary*:** *What attracted me to this program was that they promised to open accounts for us, teach us how to save. Also, they told us that they would match our savings. Even if I wasn’t able to open an account with Suubi, the sessions that were delivered gave me courage*.

Frank and Annet, along with several other participants, were particularly drawn to the program’s income-generating activity component. They expressed a strong desire to learn how to start small-scale income-generating projects, with the potential to earn money that would be helpful in case of emergencies.

**Frank** *: I wanted to know about income-generating activities where you can get money and save and use it during emergencies”.*

**Annet**: *I was motivated to participate in the program because we were told it would train us in small income-generating activities like animal rearing.*

In addition to being excited about the opportunity to open bank accounts and learn about income generating activities, some participants were also drawn to receiving Wisepill devices that would store their medication and remind them when it was time to take it, as illustrated by Ruth, “*The other thing that motivated me was about giving us the devices to help us in taking drugs.”* For Timothy, the Wise Pill device served as a key motivating factor, particularly due to his desire to safely store his medication. As a young person, he expressed concerns about the challenges of managing his medication independently. The promise of a device that could securely store his medication and remind him when it was time to take it offered him a sense of reassurance and responsibility, *“Since we were still young, they gave us a device that stored our medicine safely because some people used to store it in cold places that weren’t suitable for the medicine.”*

### Facilitators to program attendance

Participants identified several key factors that made it easier for them to continue engaging with the program. These factors ranged from the desire to learn new knowledge, to the content of the sessions, the positive attitude of the staff, transport reimbursement, and the provision of snacks, cakes, and sodas. Additionally, support from their families played a crucial role in their sustained involvement.

***Desire to learn new knowledge.*** For many participants, the desire to acquire new knowledge strongly motivated their continued program engagement. Alex explained, *“I was already interested, but learning new things every day kept me coming back.”* Similar enthusiasm was shared by Joy and Prossy, who reported learning as their primary motivation for consistent participation. Fred had specific learning goals based on the program’s outlined components, particularly saving and income-generating activities, “*They had briefed us about the program components, including saving and income-generating activities. For me, this presented an opportunity to learn how to save and discover new methods for earning money”.*

***Relevance of the sessions content.*** When discussing the program’s content, many participants including Anna, Jessica, Jovia and Mary expressed that the cartoon sessions were especially appealing to them. These sessions provided practical, relatable strategies for addressing non-adherent behaviors, offering an engaging way to learn how to manage their challenges.

Jovia shared that the cartoon sessions were particularly helpful in reinforcing her understanding of how to take her medication properly and in learning the consequences of non-adherence. She was eager to continue attending in order to learn more, “*What made me always come to the sessions was to learn how to take well my medicine well. They showed us cartoons—one taking their medicine well and the other one badly”.* Similar to Jovia, Jessica was interested not only in the adherence lessons from the cartoons but also in learning saving techniques through the financial literacy sessions. This motivation kept her returning each day.

***Support from family members.*** The support and commitment from family members was frequently mentioned by several participants as a key factor that contributed to their continued engagement in the sessions. Sam attributed his ability to complete all the sessions to the support of his grandparent, who would remind him about the sessions and help facilitate his travel to the session location, “*There was no session I didn’t attend because by that time I had no phone and they used to call my grandparent that we need Sam at the hospital. She used to tell me and I come immediately.”* Fatimah also praised her dad for ensuring she attended the sessions, either by taking her to the meeting location himself or providing her with transport.

**Fatimah**: *They had the phone contact and they would call my father and give him the date but now they call my mother and that is what they used to get me. They would call him and I come. Sometimes my father would give me money or he takes me or I walk to meet them.*

Jonah acknowledged that, without his parents’ support, given his young age at the time, he wouldn’t have been able to commit to the sessions.

**Jonah**: *My parents made it easy because they could remind me of the time and venue for the cartoon sessions. It was mainly my parents because I was still young and they had to take me whether I wanted or not but I benefited*.

***Program Incentives.*** In addition to the support from family members, participants also highlighted the incentives provided to them as a strong motivator for continuing to attend the sessions. Specifically, many participants mentioned the transport refund, which made it easier for them to attend regularly. The reimbursement for transportation costs alleviated the financial burden of traveling to the session location, particularly for those living in distant areas or without reliable access to transport. For example, Patrick mentioned that although he lived a long distance away, his transport costs were refunded each time he attended the sessions, making it easier for him to participate regularly. While Stella shared that she would often walk to the sessions, knowing that she would receive a transport refund, which allowed her to take a boda-boda (motorcycle) back home; “*we would walk to the sessions, and they would give us transport, which helped us take a boda-boda back home”.* Jennifer appreciated the lunch and snacks that they received.

**Jennifer**: *What enabled me to come was that there was food. Every time we came, they used to give us soda and cake. They would tell us to be at the place by 9 am and we would start at like 9:30, depending on where they were coming from. But they used to prepare some breakfast for us, and at lunch, they would give us soda with our food.*

***Sense of belonging.*** One participant mentioned the need to feel a sense of belonging as one of the key factors that encouraged their continued attendance in the program. Margret, for example, shared that she needed someone to talk to beyond the people she lives with at home. She explained that during the sessions, she felt heard and understood, which was an important aspect of her participation.

**Margret:** *What enabled me is sitting and having a conversation with others. When they invited us, we were able to tell musawo (interviewer) what is bothering us. It would have been possible to speak to people at home but most of the time they mind their own business.*

***Attitude of staff and facilitators.*** Another factor mentioned by the participants was the attitude of the program staff, including both the organization staff and facilitators. They praised their discipline and the way they treated participants as equals, which increased their motivation to continue participating in the sessions. Hasifah shared the following about the organization staff:

**Hasifah:** *Although I was young but it was easy for me to attend the sessions because of the nurses (referring to ICHAD staff). They used to come while smart, happy, and I used to admire them, being like them just because they were also free. They show a lot of love to a person, they are so caring, they are always happy, and they tell you something right for your life.*

Similar comments were made about the facilitators by Linda.

**Linda:** *They would come and teach us. They knew our status but they made us forget the sadness. Even if one had a problem, once you get to them you feel relieved. And you go back when you are happy and you have understood what they taught, this made me keep coming.*

***Good planning.*** Good planning emerged as a key facilitator of program engagement. Several participants appreciated the organization’s advance scheduling, which prevented conflicts with other activities and included consistent reminders about upcoming sessions. This thoughtful approach made attendance significantly easier for many participants. For example, James highlighted how the program respected their time through proactive communication.

**James:** *They never dumped sessions on us. They would tell you the program schedule in advance which allowed us and our parents to plan travel arrangements. If I remember correctly, they scheduled sessions during school holidays when we had more free time*.

For Nathan, the advance phone calls were particularly important for maintaining attendance, *“They called us ahead of time to confirm session dates. This way, you could make sure nothing would interfere on that day”.*

### Barriers to program attendance

While most participants completed the program sessions, some reported challenges that hindered consistent attendance. These barriers included transportation costs, session scheduling conflicts, health issues, and HIV-related stigma. Additionally, caregiver involvement emerged as a complex factor—while some participants felt pressured by parents to attend, others perceived limited benefit when caregivers controlled the program’s financial incentives.

***Limited transportation.*** Despite transportation refunds being provided, distance and cost remained significant barriers for some participants, particularly those living far from session venues. Some participants missed sessions when their families couldn’t afford upfront transportation costs on certain days. For example, James reported that despite receiving advance notice about sessions, he was unable to attend on days when his father couldn’t provide transportation to the venue, “*Sometimes sessions were held at Kitanda (nearby), but other times at Bigasa (far away). When my father wasn’t available to give me a ride, getting to the venue became difficult.”* Similarly, Robinah described her experience: *“Transport was usually the issue. When I was staying with my mother at my grandparent’s place, she often couldn’t afford the fare, so I missed some sessions”.*

***Timing of the sessions versus other commitments.*** Some participants mentioned that the timing of the sessions, given their other commitments, prevented them from engaging in some of the sessions. Although the sessions were scheduled during school breaks (either during break or lunch), weekends, or holidays, and the dates were communicated to parents in advance, some participants were still unable to attend due to other commitments or circumstances. For instance, Jamirah had to help with house chores first, and by the time she got to the venue, the session had already ended. Similarly, Mathias mentioned that he had to miss some sessions because he had other commitments, “*Sometimes I didn’t have the time to attend, and I would miss some sessions. There was a time when I was tied up and needed to be somewhere else… and I couldn’t find time to attend the session.”*

***Health issues.*** Furthermore, health issues, such as illness, were a barrier to session attendance. Since both the participants and their caregivers were required to attend together, if either one was sick, the session had to be missed. Aminah had to miss the sessions on the days when she was sick, while Alex mentioned that on the days his grandparent fell ill, he too had to miss the session: *‘Granny had those days when she was weak, and because there was no other adult in the house, I had to miss* the sessions.

***HIV-Related stigma***. While reported by only one participant, HIV stigma significantly impacted participation. Ritah avoided sessions due to fears of status disclosure in her community, *“The sessions were held in the community, so I worried people might see me attending and realize I’m HIV-positive—something I desperately wanted to hide. This fear made me hesitate to come”.*

***Caregiver involvement.*** While caregiver participation was required, some youth perceived it as a barrier to engagement. Program rules mandated adolescent participation with a household adult (18 + years). However, two participants reported that parental involvement in sessions hindered their attendance. For example, Jamir described how parental pressure affected him, *“I didn’t like attending with my parent —whenever I was quiet, they would question me. I ended up skipping sessions.”* While Steven disengaged when his grandparent controlled the incentives, *“I stopped coming because Granny kept all the money we received without giving me anything. I got discouraged from attending the sessions”.*

### Perceived impact

Participants’ engagement and outcomes often depend on how relevant they find the content and how they perceive the impact of these sessions on their lives. Participants found the content covered in both FLT & IGA and cartoon sessions very relevant and helpful. For instance, Jalia and many other participants appreciated the cartoon sessions for helping them adhere to their medication. When asked how helpful the sessions were, she mentioned that they were easy to understand, and the use of relatable characters and scenarios made it easier for them to connect with their treatment plans and stay committed to their health journey. Susan also realized through these sessions that adhering to her medication was a powerful tool for avoiding self-discrimination, as people wouldn’t notice that she was different from them.

**Susan:** *What I liked most were the lessons from Kamperempe (cartoon character). I learned not to self-discriminate. I have to prove that I’m not any different from the rest of the people, and that I care enough about my health. I have to adhere to my medication in order to fulfill my future prospects”.*

Similar sentiments were shared by James, who appreciated seeing the difference between those who adhered to their treatment versus those who did not.

**James**: *What I liked most about the cartoons is that you could see those who don’t adhere very well to their treatment and those who do. Depending on their appearance, one could easily identify a person who is not adhering well to treatment. We could easily spot them by their unhealthy looks. You could look at those who are happy and think, ‘Eh… I should be happy just like them.’ If you look at the smiling emoji on the viral load results, you’d also feel happy.*

In addition to improving her adherence, Esther was grateful for the cartoon sessions because they helped protect her from unwanted pregnancies and prevented her from engaging in other sexual risk behaviors, enabling her to live a healthy life; “

**Esther:** *The sessions they gave and taught us helped me so much not to engage in sexual intercourse. The sessions with cartoons, where one person takes their medicine properly and the other doesn’t. Now the other one goes with groups and the other one doesn’t, which means that if you don’t take your medicine properly and engage in sexual risk behaviors, you will be infected with other diseases and could end up with an unwanted pregnancy.*

Zaufah highlighted the value of these sessions in helping her avoid other risk behaviors, such as drinking alcohol and using drugs, as these can affect the effectiveness of the medication. She emphasized that she has carried this knowledge into adulthood.

**Zaufah**: *They asked us questions like, ‘Have you ever used alcohol or drugs?’ and explained why alcohol is harmful for people on ART. They taught us that even as adults, we need to limit drinking. They gave us so much guidance – warning us against alcohol, drugs, and other risks. It was really thorough!*

For Jonah, understanding that taking HIV medication without using protective measures such as condoms could lead to acquiring other sexually transmitted diseases, he still praised Suubi (the program) for enlightening him on this, “*We were told that if you engage in sexual behaviors without protection, you will acquire other sexually transmitted diseases, and then you end up combining two diseases, which could affect your health or lead to health deterioration.”*

Jemimah and Robert commended the program for providing them with the wise pill devices, which not only improved their medication adherence but also helped them store their drugs safely. Jemimah acknowledged that, because they were young, the device helped them store their medicine properly, as people often store it in places that are not suitable environments for such drugs. The reminders from the device also helped her adhere to the treatment. According to Robert, if it were not for the device, his viral load would not have been suppressed.

**Robert:** *I thank Suubi because in the first year, I didn’t take my medicine regularly, and my viral load was very high. Then, you came with a toolbox (the wise pill device). If I hadn’t joined this program and received the pill device, I would have died then.*

Another component of the program frequently mentioned by participants was economic empowerment, particularly learning how to save, and start income-generating activities. Frank, for instance, did not know that one could save money in a bank to address emergencies and future needs. He learned this through the program.

**Frank:** *When I was young, I had no idea that a person could save money for herself to help with future needs. I thought gangsters would steal my money, so I didn’t save it. At that time, I didn’t have this idea in my head, but my mum used to tell me to bring the money so we could save it. I thought I would never grow up and would stay young forever. However, as they continued to teach me, I came to realize that saving is important. Now that I’m older, I understand how helpful it can be, something I didn’t know when I was young*.

In addition to saving, Jacob valued the knowledge he gained about income-generating activities. To him, these projects provided another way to save money and reduce expenditure while increasing returns.

**Jacob:** *With income-generating activities and savings, I learned that you could keep your money through animal husbandry. But if you just keep it in a bank, you might end up spending it all. However, with animal husbandry, you can also make a profit.*

## Discussion

This study qualitatively explored the acceptability of the Suubi+Adherence intervention, a combination intervention among adolescents living with HIV in Southern Uganda. Specifically, the in-depth interviews explored the participants’ expectations that influenced their decision to join the study and engage in the program, the facilitators and barriers to their participation, and their views on the relevance of the program content and its perceived impact, which are important for understanding the overall acceptability of the intervention.

The participants highlighted several aspects of the program that motivated their decision to participate, including the opportunity to hold bank accounts in their names and use Wisepill devices to enhance their adherence behaviors. However, the most frequently emphasized component was the savings and income-generating activities. The emphasis on economic opportunities may be explained by the context of the study, which was conducted in one of the poorest regions of the country, characterized by high HIV prevalence [46]. In such settings, economic advancement opportunities are limited, making programs that provide savings options or teach income-generating skills especially attractive to people who may have few alternatives for financial support or employment [47]. According to the TFA, individuals are more likely to engage in programs when they feel it is more acceptable and beneficial—anticipated affective attitude [45]. Previous studies have found similar motivations among adolescents in low-resource settings. For example, Sensoy and colleagues identified the desire to learn about savings and income-generating activities as key motivators for adolescent girls in Ghana and Uganda to participate in such programs [25,26]. These findings suggest that the prospect of financial

independence is a powerful tool for youth in economically vulnerable regions.

When we explored the facilitators of program attendance, one of the most commonly mentioned factors was the strong desire to acquire new knowledge among the participants. Nearly every participant mentioned their enthusiasm for learning new skills and concepts, particularly those related to savings and income-generating activities. This desire was also mentioned by participants in Tracy’s study among adolescent girls and young women in South Africa, where the opportunity to learn new knowledge kept them motivated to attend the next sessions [48]. In addition, some participants expressed that a sense of belonging was a key facilitator to their participation in the program. For these participants, the program provided not only the opportunity to learn new skills and knowledge but also a supportive community where they felt accepted and valued. This environment offered them a space to build new friendships and connect with other adolescents facing similar challenges, which helped them feel less isolated [49,50].

Another facilitator was the support from family members. This support manifested in various forms, from providing transportation to the session venues to actively participating in the sessions. For many participants, such involvement was a strong motivator, reinforcing their commitment to the program and fostering a sense of shared responsibility for their continued engagement, which aligns with findings from other studies [51,52]. However, while some participants found this support encouraging, others perceived it as intrusive, contributing to their minimal participation in the program. Nevertheless, these findings emphasize the role of family support in sustaining youth engagement in intervention programs, especially in low-resource settings. When family members, particularly parents, are actively involved in the process—whether through logistical support like transportation or direct participation in sessions—it encourages the youths to stay engaged and strengthens the sense of accountability and shared responsibility.

Furthermore, the participants greatly appreciated the provision of incentives, including transport refunds, lunch, cakes, and sodas, which they identified as key facilitators for their continued engagement in the program. The transport refund was frequently mentioned among these, as many participants had to travel significant distances to attend the sessions. Some participants indicated that, with the expectation of reimbursement, they could borrow money to cover the travel costs, reflecting the perceived value of the intervention [45]. Providing resources for travel has been documented as a strategic approach to keep individuals motivated to participate in programs, particularly in settings where transportation costs and accessibility are significant barriers [53,54]. While the transport refund helped ease the financial burden for many, it was not always sufficient, especially for participants living in more distant areas. Even with the reimbursement, some participants still faced challenges attending all sessions, highlighting the continued barriers posed by long travel distances, as noted in previous studies [25,55].

Finally, the relationship between the program staff and participants was a crucial aspect that contributed to the participants’ continued attendance and engagement in the program. Many participants described the facilitators and program staff as approachable, supportive, and empathetic, which created a positive and trusting environment. This strong, supportive relationship helps to foster a sense of safety and comfort for participating individuals as they feel valued and heard [53,56]. As a result, they were more likely to attend the sessions regularly and remain engaged throughout the program.

In addition to parental involvement and transport costs, which hindered participants from attending all the scheduled sessions, other obstacles were also identified. These included the timing of the sessions in relation to other personal commitments, fear of being exposed, and health issues. These factors have been identified in previous studies. For instance, Sensoy et al. (2022) and Tracy et al. (2021) reported that personal commitments, including family responsibilities, interests, or activities, as well as competing responsibilities, were significant barriers to participation in programs [26]. Similarly, Ilvig et al. (2018) identified long distances, which required participants to incur additional transport costs, and illness as attendance barriers in their study [57].

The participants found the content delivered during the intervention to be highly relevant. Content relevance, in this context, refers to how well the materials, topics, and activities align with participants’ real-life experiences, challenges, and needs [58]. Many participants shared that the topics covered were practical and directly addressed their challenges. For example, the cartoon sessions focused on medication adherence, using relatable examples; the income-generating activity (IGA) sessions inspired them to explore ways of starting their own businesses; and the financial literacy sessions introduced them to saving behaviors. Previous studies have also emphasized content relevance as a key factor in maintaining participant engagement [52,59]. While some participants noted that the skills did not always translate seamlessly to real-life situations, many others emphasized their ability to apply these skills in their daily lives, demonstrating their self-efficacy in using what they had learned [45]. However, some also mentioned that being young at the time the intervention was delivered, they could hardly relate to the information at first, making it more challenging to fully engage with the content. Despite this, the benefits noted by participants strongly reflect the perceived effectiveness of the intervention, another key aspect within the TFA. When asked about their motivation for participating in the program, most participants cited a desire to gain new knowledge, excitement about savings and income-generating activities, and the use of Wisepill devices to improve medication adherence. These changes were mentioned by many participants upon completing the program and continue to be applied in their daily lives. This suggests that the intervention met their expectations in terms of perceived effectiveness [45].

Overall, our findings suggest that the participants found the Suubi+Adherence intervention highly acceptable, an important and promising step for its uptake and scalability in the region and beyond, while also pointing to certain barriers that need to be taken into account. However, study results need to be interpreted in light of study limitations. The TFA was used at the stage of data analysis, rather than at the stage of developing the interview protocol. Relatedly, certain constructs such as ethicality and intervention coherence were not explored in-depth during data collection. In addition, the data were collected retrospectively, requiring participants to recall experiences from several years earlier. This introduces the possibility of recall bias, as participants may not have accurately remembered or may have forgotten aspects of the intervention and their associated experiences. Finally, future research should examine the acceptability of the intervention from the perspectives of the caregivers and intervention facilitators as these would have implications for intervention sustainability. Despite these limitations, these findings contribute to the scarce literature on intervention acceptability among adolescents in SSA. Another strength of the study is the sampling strategy that allowed us to capture the experiences of both those who fared well on the targeted intervention outcomes and those who did not.

## Conclusion

Guided by the TFA, our results suggest that the youths found the intervention highly acceptable and that it addressed some of the challenges faced by their families in the study region, particularly through the matched savings component and income-generating activities. Hence, our findings provide valuable insights into the acceptability of a combination intervention among youths, as well as its potential for sustainability and scalability. These results also carry important programmatic and policy implications for Uganda, particularly in light of the country’s high HIV prevalence, which is even more pronounced in the study region. Therefore, it is important to consider incorporating evidence-based interventions with demonstrated acceptability and effectiveness into national HIV prevention programs and policies.

## Abbreviations

SSA: Sub-Saharan Africa
ART: Antiretroviral therapy
YLHIV: Young people living with HIV
ALHIV: Adolescents living with HIV
LMICs: Low- and middle-income countries
CDA: Child Development Account
FLT: Financial Literacy Training
IGA: Income-generating Activities
GCP: Good Clinical Practice
CITI: Collaborative Institutional Training Initiative
TFA: Theoretical Framework of Acceptability
